# Multiple primary malignancies in 788 Chinese patients with diffuse large B‐cell lymphoma

**DOI:** 10.1002/cam4.6070

**Published:** 2023-05-21

**Authors:** Xuchang Zhang, Hanning Tang, Yi Miao, Yi Xia, Huayuan Zhu, Li Wang, Lei Fan, Wei Xu, Jianyong Li

**Affiliations:** ^1^ Department of Hematology the First Affiliated Hospital of Nanjing Medical University, Jiangsu Province Hospital Nanjing China; ^2^ Key Laboratory of Hematology of Nanjing Medical University Nanjing China; ^3^ Collaborative Innovation Center for Cancer Personalized Medicine Nanjing China; ^4^ Pukou CLL Center Nanjing China; ^5^ Geriatric Department of The Second Affiliated Hospital, School of Medicine The Chinese University of Hong Kong, Shenzhen & Longgang District People’s Hospital of Shenzhen Shenzhen China

**Keywords:** diffuse large B‐cell lymphoma, incidence, multiple primary malignancies, prognosis

## Abstract

**Background:**

Diffuse large B‐cell lymphoma (DLBCL) is the most common histological subtype of non‐Hodgkin lymphoma worldwide. The emergence of multiple primary malignancies (MPMs) has been described as a new prognostic factor in many types of tumors.

**Methods:**

To investigate the morbidity, incidence, and survival of MPM in DLBCL, we retrospectively reviewed the characteristics of 788 patients with DLBCL.

**Results:**

Forty‐two patients were diagnosed with MPM, and 22 of them were diagnosed with subsequent primary malignancies (SPM) by pathologic biopsy. The incidence of SPM was associated with older age. Germinal center B‐cell‐like (GCB) subtype and earlier Ann Arbor stage DLBCL patients were more prone to SPM. MPM, age, lactate dehydrogenase (LDH) level, Eastern Cooperative Oncology Group performance status (ECOG PS), Hans classification, and international prognostic index (IPI) score were prognostic risk factors for overall survival (OS).

**Conclusion:**

These data provide a comprehensive view of MPM in DLBCL. MPM was an independent prognostic factor of DLBCL in univariate analysis.

## 1 INTRODUCTION

Malignancy, a significant disease in global society, has become the second largest cause of death in Americans and the leading cause of death for Chinese residents since 2010.^1^, ^2^ The incidence of malignant tumors has been increasing recently, although the survival rates of some common malignant tumors have also been increasing yearly. However, a great number of these survivors are at long‐term risk of subsequent primary malignancies (SPMs), and the increased incidence of SPM is a well‐known late effect of tumors, thereby raising research hotspots in the risk factors, pathogenesis, and prognostic factors of multiple primary malignancies (MPMs).^3^ New SPM patients account for approximately 17% of all new cancers each year, according to the national cancer surveillance institute.^4^ Long‐term studies on survivors of hematologic malignancy show that SPM is becoming one of the major causes of death for these survivors.^5^

The incidence of non‐Hodgkin's lymphomas (NHLs) has been increasing in most of the Western countries. Recent advances in medicine, especially monoclonal antibodies such as rituximab, have improved the prognosis of patients with diffuse large B‐cell lymphoma (DLBCL), the most common histological subtype of NHL.^6^ Therefore, many patients with DLBCL become long‐term survivors, and the risk of SPM has become a hotspot of international research. In China, DLBCL is also the most frequent subtype of NHL, but few studies have focused on MPM in DLBCL. This study not only focused on exploring the prognostic role of a history of MPM in Chinese DLBCL patients but also concentrated on the time and risk of SPM after DLBCL. Hence, we retrospectively investigated the clinical characteristics of MPM to create a better understanding of the prognostic prediction of the MPM history and the incidence of SPM in DLBCL patients.

## 2 MATERIALS AND METHODS

### 2.1 Diagnostic Criteria

All of the patients were diagnosed by MPM criteria: (i) tumors have definite features of malignancy; (ii) tumors have unique pathological characteristics; and (iii) the possibility of a tumor being a metastasis of the index tumor is ruled out.^7^ Past cancer is defined as patients with preceding cancer(s) that occurred more than 2 months before the diagnosis of DLBCL, and synchronous cancer refers to patients diagnosed with DLBCL and other malignancies within 2 months.^8^ Patients detected to have another malignancy 2 months after DLBCL diagnosis are considered to have SPM.^9^

### 2.2 Patients

In total, 788 DLBCL patients diagnosed from June 2008 to June 2019 in our hospital were enrolled in this study, and patients who missed their follow‐up appointment were excluded. According to diagnostic criteria, the patients were classified into three groups: 20 with MPM history, including 14 with past cancer and six with synchronous cancer; 22 with SPM; and 750 without another cancer. All patients were confirmed by histopathology or cytopathology, and HIV‐positive patients were excluded from the retrospective study. This study was approved by the hospital ethics committee, and all patients provided informed consent according to the Declaration of Helsinki.

### 2.3 Data collection

Detailed clinical features, laboratory data, and survival data of these patients were collected in our study. Patients' basic data, initial disease staging, pathological and laboratory results, prognostic factors, treatment status, and MPM‐related data were collected from the hospital‐based service and follow‐up by telephone.

### 2.4 Statistical analyses

SPSS 20 (IBM Corporation.) and GraphPad Prism 6.0 were used to analyze the data. Categorical variables were analyzed by χ2 test. Methods were selected according to the following principles: (1). For total count ≥40 and minimum expected count ≥5, we used the Pearson chi‐squared test. (2). For total count ≥40 and 1 ≤ minimum expected count <5, we used continuity correction. (3). For total count <40 or minimum expected count <1, we used Fisher's exact test. Overall survival (OS) was defined as the time from diagnosis of the first tumor to death or last follow‐up. Survival curves were constructed by the Kaplan–Meier method, and the log‐rank test was used for statistical associations. The Cox proportional hazards model was established to evaluate the effect of different factors on survival and hazard ratio (HR) by multivariate analysis. The results were evaluated with 95% confidence intervals (CIs), and the significance level was set at two‐sided *p* < 0.05.

## 3 RESULTS

### 3.1 Characteristics of DLBCL patients

In this study, 788 DLBCL patients were included, and 42 patients were diagnosed with MPM. The ratio of males/females was 1.4. The median age was 66 years old. In total, 20 patients were diagnosed with MPM at the baseline of DLBCL. Six MPM patients were diagnosed with other primary malignancies within 2 months after DLBCL, and primary malignancies were identified in the remaining 14 patients before DLBCL diagnosis (Table 1 and Table 2). During the follow‐up period, 202 (25.5%) patients died, and 22 (2.86%) had SPM. The mean interval from diagnosis of DLBCL to SPM was 37.32 months (3–108 months). There were four patients' SPMs in 1 year, 14 patients' SPMs from 1 to 5 years and four patients' SPMs 5 years later. According to the location of SPM, eight cases occurred in the respiratory system (lung cancer), four cases in the digestive system, four cases in the blood system, two cases in the urogenital system, one case papillary thyroid carcinoma, one case basal cell carcinoma of the left inner canthus, and one case endometrial adenocarcinoma (Table 3). Table 2 Patient No. 3 was successively diagnosed with (1). Gastric cancer (subtotal gastrectomy); (2). Esophageal cancer (surgery and radiotherapy); (3). Skin cancer (surgical treatment); (4). DLBCL (nongerminal center B‐cell type), stage IV Group A, Eastern Cooperative Oncology Group performance status (ECOG PS) 3, international prognostic index (IPI) score 5 (4 cycles of chemotherapy with “R + GemOx”, 4 cycles of chemotherapy with “R + miniCHOP”) and died 1 year after diagnosis of DLBCL. Another case of Table 2 No. 5 was diagnosed as follows: (1). Papillary thyroid carcinoma (surgical treatment); (2). DLBCL, stage III Group A (nongerminal center B‐cell type, aaIPI score 1, ECOG PS 0, six cycles of R‐CHOP chemotherapy); (3). Clear cell renal cell carcinoma, stage II (surgical treatment), and is still alive.

**TABLE 1:** Characteristics of 6 synchronous cancer group patients with DLBCL.

Number	Gender	Age (Year)	Consolidated tumors	Status	OS (Months)
1	Male	54	Clear cell renal cell carcinoma	Survival	40
2	Male	66	High‐grade intraepithelial neoplasia (carcinogenesis) of esophageal squamous epithelium	Survival	38
3	Male	53	Myelodysplastic syndrome	Survival	21
4	Male	72	Colon adenocarcinoma	Die	13
5	Male	77	Renal cancer	Die	4
6	Male	84	Myelodysplastic syndrome	Die	2

Abbreviations: DLBCL, diffuse large B‐cell lymphoma; OS, overall survival.

**TABLE 2:** Characteristics of 14 past cancer group patients with DLBCL.

Number	Gender	Age(year)	1st Tumor	Interval time between the first tumor and DLBCL (Months)	Status	OS (Months)
1	Female	44	Breast carcinoma	120	Survival	4
2	Female	52	Liver cancer	39	Survival	18
3	Female	53	Gastric carcinoma	240	Die	12
4	Male	54	Middle esophageal squamous cell carcinoma	28	Survival	71
5	Female	57	Papillary thyroid carcinoma	29	Survival	44
6	Female	62	Liver cancer	117	Survival	9
7	Male	64	Rectal ulcerative adenocarcinoma	20	Die	1
8	Female	66	Colon adenocarcinoma	70	Survival	15
9	Male	68	Esophagus cancer	240	Survival	28
10	Female	69	Breast carcinoma	480	Survival	10
11	Female	69	Endometrial cancer	203	Survival	1
12	Male	76	Vocal cord squamous cell carcinoma	252	Die	1
13	Male	80	Prostate cancer	72	Die	12
14	Female	87	Breast carcinoma	180	Die	95

Abbreviations: DLBC, diffuse large B‐cell lymphoma; OS, overall survival.

**TABLE 3:** Characteristics of 22 SPM group patients with DLBCL.

Number	Gender	Age (Year)	Interval time between DLBCL and SPM (Months)	Diagnosis of SPM	Status	OS (Months)
1	Male	50	3	Enteropathy‐related T‐cell lymphoma	Die	20
2	Male	67	5	Lung adenosquamous carcinoma	Die	5
3	Female	66	7	Pancreatic cancer	Die	9
4	Female	49	8	Papillary thyroid carcinoma	Survival	30
5	Male	63	13	Renal cell carcinoma	Die	20
6	Male	73	14	Gastric adenocarcinoma	Die	27
7	Male	75	15	Gastric antrum adenocarcinoma	Die	24
8	Male	68	17	Lung adenocarcinoma	Survival	28
9	Female	75	25	Lung invasive adenocarcinoma	Survival	37
10	Female	76	30	Colon adenocarcinoma	Die	75
11	Male	55	35	Clear cell renal cell carcinoma	Survival	73
12	Male	71	36	Lung adenosquamous carcinoma	Survival	65
13	Female	58	36	Lung adenocarcinoma	Survival	102
14	Male	26	39	Myelodysplastic syndrome	Survival	51
15	Male	73	49	Basal cell carcinoma of the left inner canthus	Survival	72
16	Female	71	56	Acute non‐lymphocytic leukemia‐M2	Die	60
17	Male	84	58	Lung cancer	Die	62
18	Female	63	60	Endometrial adenocarcinoma	Survival	102
19	Male	66	63	Lung adenocarcinoma	Survival	74
20	Male	77	64	Colon adenocarcinoma	Survival	130
21	Female	81	80	Lung adenocarcinoma	Survival	98
22	Female	65	108	Myelodysplastic syndrome	Die	110

Abbreviations: DLBCL, diffuse large B‐cell lymphoma; SPM, subsequent primary malignancy; OS, overall survival.

### 3.2 Correlation between SPM incidence and other factors

This study focused on the relationship between the incidence of SPM and the baseline clinical characteristics and treatment strategy of patients, such as sex, age, IPI score, Hans classification, and utilization of rituximab. Patients over 60 were more inclined to suffer from SPM (*p* = 0.010). In addition, the germinal center B‐cell‐like (GCB) subtype and earlier stage were more prone to SPM (*p* = 0.056), although this difference was not statistically significant. There was no significant correlation between SPM and sex, Ann Arbor stage, extranodal involvement, IPI score, B symptoms or utilization of rituximab (Table 4).

**TABLE 4:** Risk profiles of SPM in 768 patients with DLBCL.

Characteristics		Not Merge SPM (Number)	Merge SPM (Number)	OR (95% CI)	*p*‐Value
Gender	Male	398	13	0.792 (0.334–1.875)	0.595
	Female	348	9		
Age	≤60	410	6	3.254 (1.259–8.408)	**0.010**
	>60	336	16		
Stage	1–2	289	13	0.441 (0.186–1.044)	**0.056**
	3–4	454	9		
Number of extranodal involvements	<2	531	12	2.058 (0.876–4.835)	0.091
	≥2	215	10		
IPI score	0–2	526	13	1.534 (0.627–3.755)	0.345
	3–5	211	8		
With B symptoms	No	510	16	0.810 (0.313–2.097)	0.664
	Yes	236	6		
Hans classification	Non‐germinal center type	456	9	2.271 (0.959–5.381)	**0.056**
	Germinal center type	290	13		
Utilization of Rituximab	No	57	2	0.827 (0.189–3.629)	0.683
	Yes	689	20		

The significance of *p* < 0.05 are indicated in bold.

Abbreviations: CI, confidence interval; DLBCL, diffuse large B‐cell lymphoma; OR, odds ratio; SPM, subsequent primary malignancy.

### 3.3 Incidence of SPM in DLBCL and clinical outcome

**FIGURE 1:**
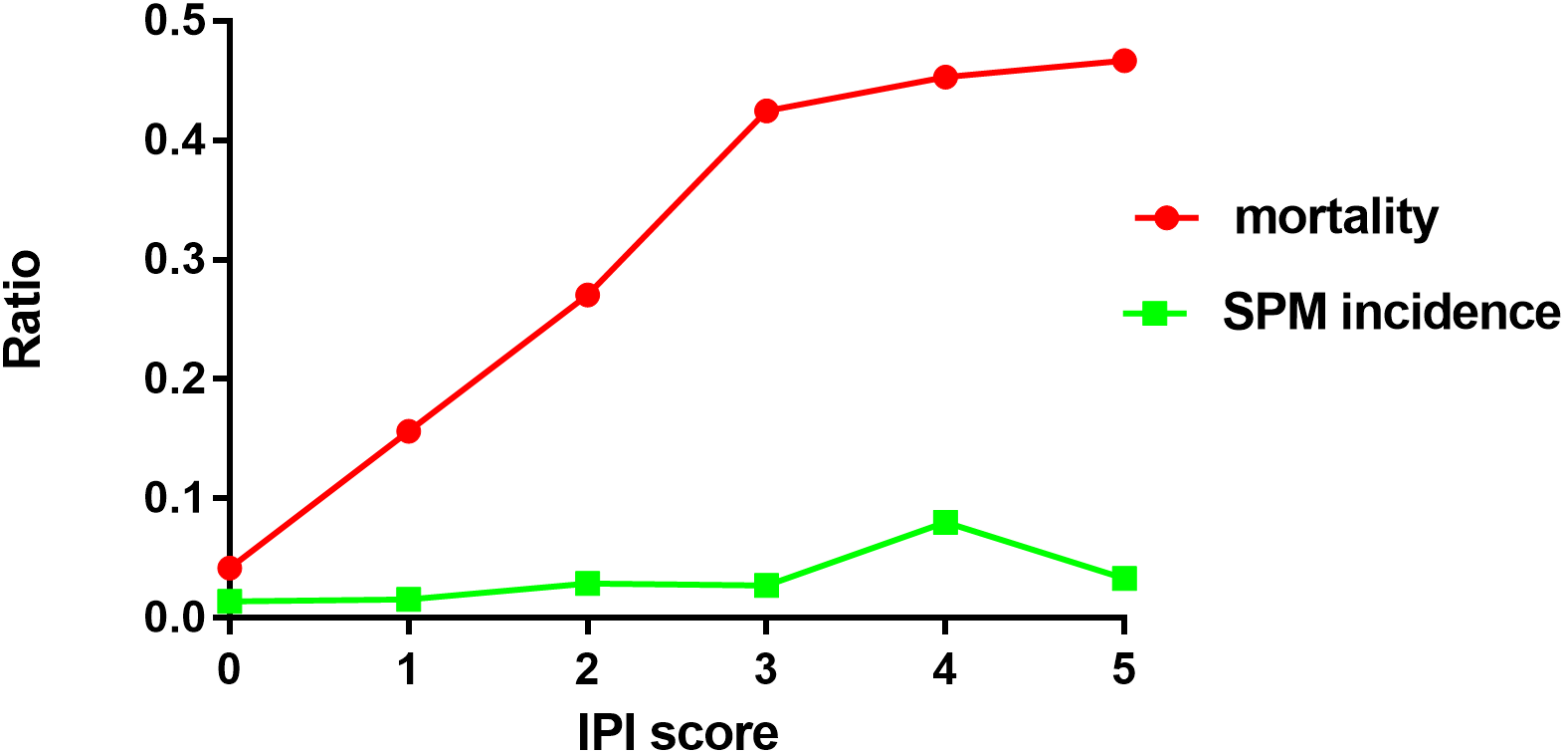
The relationship between mortality, SPM incidence and IPI score. IPI, international prognostic index; SPM, subsequent primary malignancy.

**FIGURE 2:**
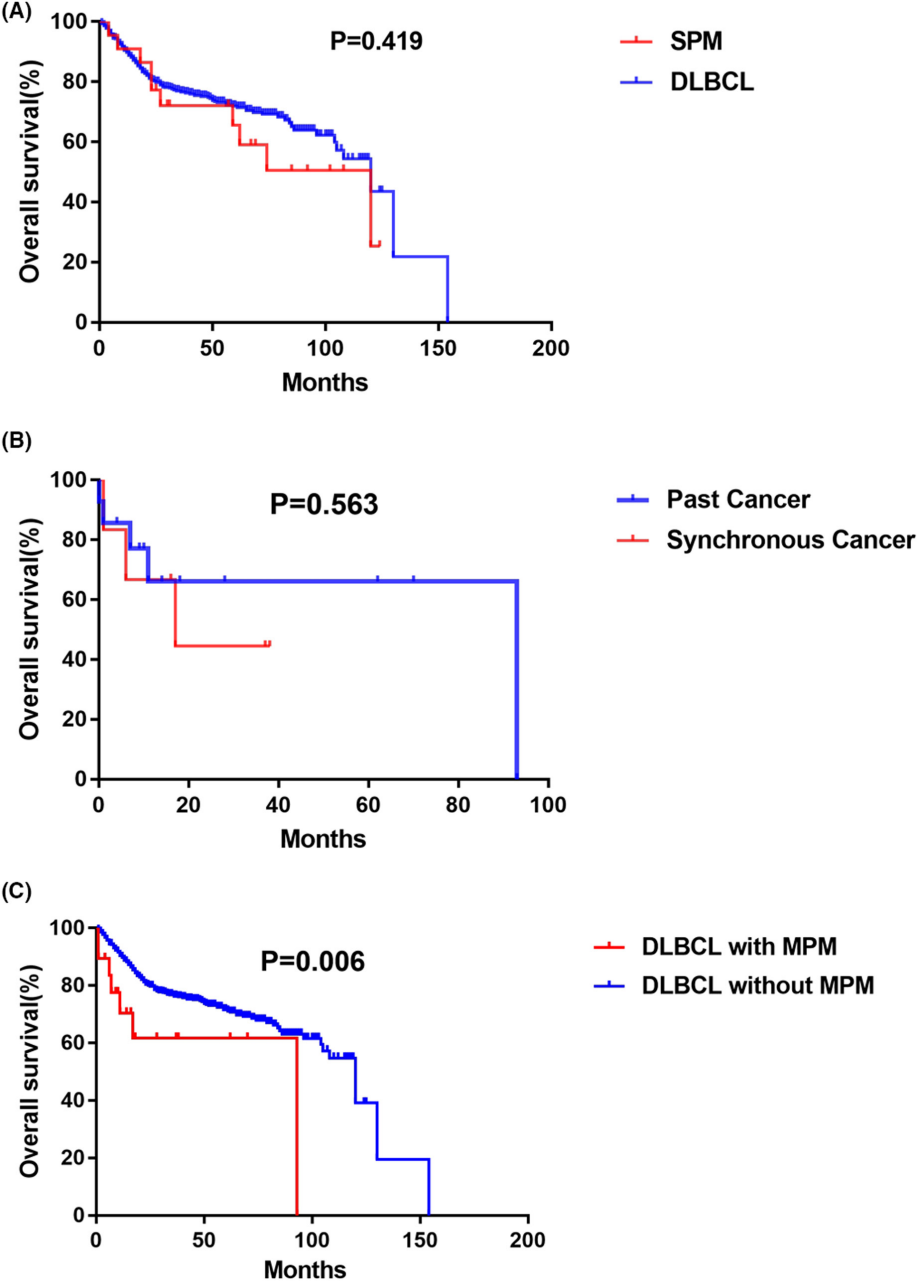
Kaplan–Meier analysis curves of overall survival from for SPM group and DLBCL group (A). Kaplan–Meier analysis curves of overall survival for past cancer subgroup and synchronous cancer subgroup (B). Kaplan–Meier analysis curves of overall survival for MPM group and DLBCL group (C). DLBCL, diffuse large B‐cell lymphoma; MPM, multiple primary malignancy; OS, overall survival; SPM, subsequent primary malignancies.

All 788 DLBCL patients were followed up for 10 years. We investigated the incidence of SPM in 768 patients with single DLBCL. Although there were no significant correlation between IPI scores and SPM, the incidence rate of SPM increased with increasing IPI scores (Figure 1). The OS of SPM patients and single DLBCL patients was not significantly different (5‐year OS: 65.6% vs. 72.6%, *p* = 0.419). Both median OS was 120 months. However, the survival curves for OS in these groups were rarely superimposed, and the SPM patients had worse OS at most of the times in our cohort (Figure 2A).

### 3.4 Prognostic impact of MPM in DLBCL and clinical outcome

We next investigated the prognostic impact of MPM on the baseline characteristics of DLBCL patients. There were no significant differences in OS between patients with past cancer and those with synchronous cancer (Figure 2B). Given that the prognoses of patients with past cancer and synchronous cancer were statistically equivalent, these two groups were combined into one single cohort of MPM for further investigation. There was a statistically significant difference between the OS of DLBCL patients with MPM and that of DLBCL patients without MPM (5‐year OS: 58.6% vs. 71.6%, *p* = 0.006). The MPM patients had inferior OS, and the median OS was 93 and 120 months, respectively (Figure 2C).

### 3.5 Univariate and multivariate Cox regression analyses

The results of the univariate and multivariate Cox proportional hazards regression analyses are summarized in Table 5. The univariate results showed that age > 60 years (*p* < 0.001), lactate dehydrogenase (LDH) > upper limit of normal (ULN) (*p* < 0.001), ECOG PS≥2 (*p* = 0.002), IPI score ≥3 (*p* < 0.001), non‐GCB subtype (*p* < 0.001), and MPM (*p* = 0.006) had adverse impacts on OS. Multivariate Cox regression analysis showed that age > 60 years (*p* < 0.001), LDH > upper limit of normal (ULN) (*p* = 0.002), IPI score ≥3 (*p* < 0.033), B symptoms (*p* = 0.014), and non‐GCB subtype (*p* < 0.001) were associated with inferior OS. MPM (*p* = 0.442) was seemingly not associated with OS. (Table 5).

**TABLE 5:** Univariate and multivariate Cox regression analyses of OS in 766 DLBCL patients.

	Univariate analyses (OS)		Multivariate analyses (OS)	
Characteristics	HR (95% CI)	*p*‐Value	HR (95% CI)	*p*‐Value
Female	0.969 (0.726–1.294)	0.832	–	–
Age > 60 years	2.268 (1.685–3.051)	**<0.001**	1.881 (1.380–2.564)	**<0.001**
LDH > ULN	1.700 (1.271–2.275)	**<0.001**	1.599 (1.184–2.158)	**0.002**
Ann Arbor stage III or IV	0.999 (0.745–1.339)	0.993	–	–
Extranodal involvement≥2	1.297 (0.958–1.758)	**0.098**	1.088 (0.792–1.495)	0.601
ECOG PS≥2	1.657 (1.192–2.302)	**0.002**	1.386 (0.970–1.982)	0.073
IPI score ≥3	1.828 (1.361–2.454)	**<0.001**	1.414 (1.029–1.942)	**0.033**
B symptoms	1.319 (0.980–1.776)	**0.068**	1.474 (1.083–2.005)	**0.014**
GCB subtype	0.517 (0.374–0.713)	**<0.001**	0.532 (0.382–0.741)	**<0.001**
Utilization of Rituximab	0.968 (0.550–1.703)	0.91	–	–
Merge MPM	2.608 (1.283–5.303)	**0.006**	0.742 (0.347–1.586)	0.442

The significance of *p* < 0.05 are indicated in bold.

Abbreviations: CI, confidence interval; 95% CI, 95% confidence interval; DLBCL, diffuse large B‐cell lymphoma; ECOG PS, Eastern Cooperative Oncology Group performance status; GCB, Germinal center B cell‐like; HR, hazard ratio; IPI, international prognostic index; LDH, lactate dehydrogenase; MPM, multiple primary malignancies; OS, overall survival; ULN, upper limit of normal.

## 4 DISCUSSION

New generation drugs and other regimens have extended the life expectancy of patients with NHL.^10^, ^11^, ^12^, ^13^, ^14^ DLBCL is the most frequent type of NHL. Prior studies indicate that patients with NHL are at greater risk for SPM.^15^, ^16^, ^17^ This study retrospectively analyzed 788 newly diagnosed DLBCL patients, and 42 were diagnosed with MPM, of whom 22 had SPM. A prior study indicated that the incidence of SPM after DLBCL was 3.8%, which was similar to our results.^18^ According to the location of SPM, the incidence of lung cancer and colorectal cancer after DLBCL was similar to that reported previously.^9^, ^14^ Morton LM's study showed a significant increase in the incidence of melanoma after DLBCL, but no patients with melanoma were found in this study.^19^ Our results showed that lung cancer was the most common after DLBCL, which may be due to ethnic or regional differences, and the mechanism still needs further study.

However, the incidence of SPM in this study was slightly lower than that of other studies, and the tumor interval was shorter, which may be related to the fact that our observation and follow‐up time was not long enough.^14^ In addition, due to economic scarcity, patients could not afford rituximab or even the recommended course of chemotherapy. As chemotherapy reduced the immune level and prolonged the duration of rituximab‐induced B‐cell depletion and T‐cell inactivation, their immune surveillance was impaired, and the prolonged immunosuppressive status may lead to the development and progression of some SPMs.^20^, ^21^ Another notable thing is that the incidence of SPM was the highest within 5 years, accounting for 81.8%, while most studies reported an increased SPM incidence with year.^9^, ^14^ What accounts for this might be the relatively shorter follow‐up. Myeloid neoplasm showed a more significantly increased incidence than other solid tumors, and a SPM patient with myelodysplastic syndrome observed in our cohort had the longest intervals (9 years) from DLBCL diagnosis. More SPM, especially myeloid neoplasm, might occur as time passes.

We assessed the risk in the SPM subgroup. The results showed that SPM was more inclined to occur in patients over 60, with GCB subtype and earlier Ann Arbor stage. Other studies show partially similar findings.^17^, ^18^ Although the difference between the IPI score and risk of SPM was not significant, the incidence of SPM increased with increasing IPI score, which was consistent with other studies.^22^ We found that GCB subtype DLBCL patients may be highly correlated with the occurrence of SPM, which has not yet been reported. However, larger studies are still needed to verify this finding and to conduct further studies. Several studies have reported an overall increased risk of SPM after NHL treatment.^14^, ^15^, ^17^ However, in most studies, all types of NHL were combined for analysis, and in this study, we analyzed the incidence and risk factors for SPM only in a large number of patients with DLBCL. A prior study indicated that genetic alterations were associated with MPM.^23^ The genome‐wide profiling could be used to identify potential driver DNA copy number alterations and somatic mutations that promote the development of MPMs in further study.

This study also evaluated the prognostic factors of MPM in DLBCL. The OS of MPM patients was significantly shorter than that of the single DLBCL group, which had been reported before.^24^ However, the multivariate Cox regression analysis presented discrepancies. Due to the different pathological features of the first tumor in the past cancer group, OS depends on the prognosis of the first tumor, and the prognosis varies greatly. In addition, we did not conduct a detailed analysis of other risk factors for MPM (different treatment regimens, smoking history, family history, etc.), which may affect the accuracy of our prognostic results, so further studies are necessary.

In conclusion, this study describes the clinical characteristics of patients with MPM complicated with DLBCL. Patients who were over 60 years, with GCB subtype and with Ann Arbor stage<3 may have an increased risk of SPM, which will provide early diagnostic clues for SPM post DLBCL, and these factors will be the focus of future monitoring of DLBCL patients. Vigilance of SPM risk factors will promote early detection and treatment of SPM and prolong the survival of patients. This study improves our understanding of the risk of SPM, which is associated with poor prognosis, and clarifies that MPM may be an independent prognostic factor of DLBCL.

## AUTHOR CONTRIBUTIONS

**Xuchang Zhang:** Conceptualization (equal); data curation (equal); formal analysis (equal); investigation (equal); methodology (equal); writing – original draft (equal). **Hanning Tang:** Conceptualization (equal); data curation (equal); formal analysis (equal); investigation (equal); methodology (equal); writing – original draft (equal). **Yi Miao:** Conceptualization (equal); formal analysis (equal); investigation (equal); methodology (equal); validation (equal); writing – review and editing (equal). **Yi Xia:** Investigation (equal); methodology (equal); resources (equal); validation (equal). **Hua‐Yuan ZHU:** Resources (equal); supervision (equal); validation (equal). **Li Wang:** Resources (equal); supervision (equal). **Lei Fan:** Resources (equal); supervision (equal). **WEI XU:** Conceptualization (equal); project administration (equal); supervision (equal); validation (equal); writing – review and editing (equal). **Jianyong Li:** Conceptualization (equal); project administration (equal); supervision (equal); validation (equal); writing – review and editing (equal).

## CONFLICT OF INTEREST STATEMENT

The authors declare no conflict of interest.

## Data Availability

Encourages Data Sharing.
